# Case reports may help bring up younger thoracic and cardiovascular surgeons as more academic surgeons

**DOI:** 10.1186/s44215-022-00001-6

**Published:** 2022-09-27

**Authors:** Yoshiki Sawa

**Affiliations:** 1 Osaka University, Suita, Japan; 2 Osaka Police Hospital, Osaka, Japan

At present, most surgical journals only rarely accept a small number of case reports because such reports seldom contribute to increasing the journal’s impact factor. However, some number of previous case reports have contributed greatly to the significant progress of medical science, especially with regard to our understanding of surgical indications and technical consideration for rare cases. Moreover, any surgeon is likely to have experienced enormously valuable cases with correspondingly amazing discoveries during the course of their daily practice. When I was a cardiovascular surgery resident, I experienced the first case of Norwood operation for a neonate with hypoplastic left ventricular syndrome in Japan and wrote the first case report of this case making it a memorable experience where I received an incredible amount of encouragement followed by a high volume of academic activity and advancement in my career. We believe that case report journals might play a significant role as a gateway for younger surgeons to become interested in training as academic surgeons.

The Japanese Association of Thoracic Surgery (JATS) publishes an international journal, *General Thoracic and Cardiovascular Surgery* (GTCS), which encourages young surgeons to submit interesting or educational case reports. Now, we have started a new case report journal called *General Thoracic and Cardiovascular Surgery Cases* (GTCC), a sister journal to complement GTCS. We welcome younger surgeons to submit case reports to JATS cases, which will be a completely open-access online journal. I anticipate that our new journal will contribute significantly to the progress of surgery and help bring up younger academic surgeons, especially in the fields of thoracic and cardiovascular surgery.

